# Phenolic Compounds of Propolis Alleviate Lipid Metabolism Disorder

**DOI:** 10.1155/2021/7615830

**Published:** 2021-02-20

**Authors:** Lingjie Kong, Yuhao Zhang, Zhouxu Feng, Jie Dong, Hongcheng Zhang

**Affiliations:** ^1^Institute of Apicultural Research, Chinese Academy of Agricultural Sciences, Beijing 100093, China; ^2^National Research Center of Bee Product Processing, Ministry of Agriculture, Beijing 100093, China; ^3^College of Food Engineering, Harbin University of Commerce, Harbin 150076, China

## Abstract

Lipid metabolism disorder is one of the significant risk factors for a multitude of human diseases and has become a serious threat to human health. The present study aimed to evaluate the effects of phenolics from poplar-type propolis on regulating lipid metabolism by using cell models of steatosis induced by palmitic acid (PA). Our study shows that phenolic esters have higher lipid-lowering activities than phenolic acids, especially for three caffeic acid esters, including caffeic acid phenethyl ester (CAPE), caffeic acid cinnamyl ester (CACE), and caffeic acid benzyl ester (CABE). Most notably, CACE presents prominent properties to prevent intracellular lipid accumulation and to amend extracellular adipokine secretion abnormalities. In addition, our results firstly reveal that CACE can alleviate lipid metabolism disorder through mediating protein kinase RNA-like endoplasmic reticulum kinase (PERK), activating transcription factor 6 (ATF6) signaling pathway-associated protein expression, suppressing endoplasmic reticulum (ER) stress, and activating peroxisome proliferator-activated receptors (PPARs) by distinct upregulation of PPAR*α* and downregulation of PPAR*γ*.

## 1. Introduction

At present, nearly one billion adults worldwide are overweight, and at least 300 million are obesity, especially in Europe, North America, and a large number of developing nations. Obesity, hypertension [1], diabetes [2], and cardiovascular disease [3] are seriously threatening human health with rising morbidity and mortality rates. Given to these health issues, many researchers have been alarmed that obesity can be driven by a high and growing prevalence of metabolic syndrome [4, 5]. Although the concept of metabolic syndrome may not reach a universal agreement yet [6, 7], there is an inextricable connection between metabolic syndrome and lipid metabolism disorder [8, 9]. To alleviate lipid metabolism disorder, many drugs have been proved to be an effective approach. For example, statin drugs, structural analogs to hydroxymethylglutaryl-coenzyme A (a precursor of cholesterol), are the most widely prescribed therapy for decreasing blood lipid levels [10, 11]. However, long-term use of these drugs can cause statin intolerance and adverse effects, such as gastrointestinal upset, chronic muscle disease, hepatitis, and liver cell damage [12, 13]. Therefore, great efforts are underway to explore beneficial components from natural products with less adverse effects and hepatotoxicity.

Humans had used bee products for thousands of years, such as honey, royal jelly, bee pollen, and propolis. These natural bee products have been regarded as folk medicine due to their extensive pharmaceutical properties [14, 15]; for example, antimicrobial [16], anti-inflammatory [17], antioxidative [18], and antineoplastic capacities [19]. Propolis is colloidal resin collected by honeybees *Apis mellifera* from tender buds or exudates of plants and mixed with beeswax and other bee secretions. In recent years, several studies have revealed its assistant therapeutic effect against lipid metabolic diseases. For instance, Li et al. reported that encapsulated propolis modulated lipid metabolism of type 2 diabetes mellitus rats by significantly inhibiting the increase of triglycerides [20]. Zhu et al. indicated that Chinese propolis extracts helped reduce total cholesterol levels of blood lipids by 16.6% in diabetic rats [21]. Nakajima et al. founded that Brazilian propolis extracts can mitigate the lipid metabolism of experimental periodontitis in mice [22]. However, these studies tended to focus on the beneficial effects of propolis extracts rather than their specific compounds.

Propolis composition is greatly complicated; for example, more than 300 components have been identified as propolis constituents from different geographical origins [23]. Poplar-type propolis is predominantly located in China, North America, and Europe. Specifically, its main constituents are phenolics, including flavonoids, phenolic acids, and esters [24]. Attributed to strong biological properties, previous research has noted that the phenolic compounds of propolis seem to play a critical role in the regulation of lipid metabolism [25, 26]. These studies only concentrated on evaluating the lipid-lowering activities of flavonoids [27, 28]. Unfortunately, little attention has been paid to the phenolic acid and esters of propolis regarding the regulation of lipid metabolism.

In the present study, we investigated the phenolic compositions of poplar-type propolis. We also examined the abilities of these phenolic compounds to inhibit intracellular lipid accumulation and improve extracellular adipokine secretion abnormalities in palmitic acid-induced cells. In addition, we proposed the possible molecular mechanisms of caffeic acid cinnamyl ester to alleviate lipid metabolism disorder.

## 2. Materials and Methods

### 2.1. Materials

Caffeic acid (CFA), *p*-coumaric acid (CMA), ferulic acid (FRA), isoferulic acid (IFRA), 3, 4-dimethoxy cinnamic acid (DMCA), cinnamic acid (CNA), 4-methoxy cinnamic acid (MCNA), cinnamylideneacetic acid (CDA), caffeic acid benzyl ester (CABE), caffeic acid phenethyl ester (CAPE), and cinnamic acid cinnamyl ester (CCE) were purchased from Sigma-Aldrich Co. (St. Louis, MO, USA). Ferulic acid benzyl ester (FABE), *p*-coumaric acid benzyl ester (CMBE), caffeic acid cinnamyl ester (CACE), 4-methoxy cinnamic acid cinnamyl ester (MCC), and *p*-coumaric acid cinnamyl ester (CMCE) were collected from propolis by preparative HPLC with purity 95%. Bicinchoninic acid (BCA) protein assay kit, dimethyl sulfoxide (DMSO), 3-(4,5-dimethyl-2-thiazolyl)-2,5-diphenyl-2-H-tetrazolium bromide (MTT), palmitic acid (PA), fenofibric acid (FFBA), tauroursodesoxycholic acid (TUDCA), Oil Red O, paraformaldehyde, hematoxylin, isobutyl methyl xanthine (IBMX), dexamethasone, and insulin were obtained from Sigma-Aldrich (St. Louis, MO, USA). Fetal bovine serum (FBS), trypsin-EDTA solution 1× (0.25% trypsin, 0.02% EDTA), penicillin-streptomycin (P/S), nonessential amino acids, and fatty acid-free bull serum albumin (BSA) were obtained from Gibco (Grand Island, NY, USA). Dulbecco's modified eagle medium (DMEM) and phosphate-buffered saline (PBS) were from Solarbio (Beijing, China). Multifactor assay kits containing adiponectin, interleukin-6 (IL-6), leptin, monocyte chemoattractant protein-1 (MCP-1), resistin, plasminogen activator inhibitor-1 (PAI-1), and tumor necrosis factor-alpha (TNF-*α*) was purchased from Merck Millipore (Darmstadt, Germany). *β*-Actin antibody, CCAAT/enhancer-binding protein homologous protein (CHOP) antibody, activating transcription factor-6*α* (ATF6*α*) antibody, inositol-requiring protein-1*α* (IRE1*α*) antibody, activating peroxisome proliferator-activated receptor-*α* (PPAR*α*) antibody, PPAR*β*/*δ* antibody, and PPAR*γ* antibody were purchased from Abcam (Cambridge, MA, USA). Anti-mouse and anti-rabbit antibodies conjugated to horseradish peroxidase were obtained from Solarbio (Beijing, China). RIPA lysis buffer and phenylmethylsulfonyl fluoride (PMSF) were purchased from Beyotime (Nanjing, Jiangsu, China). Ethanol and isopropanol (Analytical reagent grade) were purchased from Beijing Chemical Works (Beijing, China). Methanol and acetic acid in HPLC grade were obtained from Fisher Scientific (Pittsburgh, PA, USA). Ultrapure water was purified by a Milli-Q-Integral System (Merk Millipore, MA, USA).

### 2.2. Preparation of Propolis Ethanolic Extracts

Propolis samples in this study were collected by professional beekeepers from 15 hives. These hives were located in five provinces, and each province collected three hives, including Anhui, Hubei, Hunan, Shandong, and Zhejiang provinces in China. This raw propolis was stored in a refrigerator at -18 °C until analysis.

Raw propolis (0.5 g) was cut into small pieces and extracted with 10 mL 75% ethanol solvent (ethanol/water, v/v) for 3 h at room temperature using an ultrasonic extractor at 40 kHz, 100 W. The extracts were then centrifuged to remove the residual solids, and the supernatant was filtered through a 0.22 *μ*m filter for subsequent HPLC analysis.

### 2.3. Phenolic Composition of Propolis Ethanolic Extracts by HPLC Analysis

To analyze the phenolic composition of propolis, the supernatant above mentioned was determined by LC-6AD chromatograph (Shimadzu, Tokyo, Japan) equipped with a photodiode array detector. All separations were achieved on an analytical reversed-phase column Gemini C_18_ (150 × 4.6 mm, 3 *μ*m) (Phenomenex, Inc., CA, USA). The LC mobile phase consisted of 2% acetic acid water (A) and 2% acetic acid methanol (B). A gradient program was performed with a flow rate of 0.65 mL/min, as follows: 22%–32% B (0–10 min), 32%–35% B (10–25 min), 35%–38% B (25–35 min), 38%–51%B (35–52 min), 51%–52% B (52–70 min), 52% B (70–80 min), 52%–53% B (80–90 min), 53%–59% B (90–100 min), 59%–63% B (100–115 min), 63%–75% B (115–130 min), and 75%–80% B (130–150 min). The elution of the compounds was monitored at a wavelength of 280 nm. The assignment of peaks of chromatogram was performed by comparing the retention time and UV spectra with authentic standards. And the peak areas were measured for quantitative analysis by external calibration curves.

### 2.4. Cell Culture

Human liver cell line (L02) and mouse preadipocyte line (3T3-L1) were obtained from the cell bank of the Chinese Academy of Sciences (Beijing, China). All cell lines were cultured in a DMEM medium containing FBS (10% v/v), 1% antibiotics (100 *µ*g/mL penicillin streptomycin) and 1% nonessential amino acids. The cells were maintained at 37°C in a humidified air atmosphere with 5% CO_2_.

### 2.5. Cell Viability Assays

Cell viability was determined by MTT assay. Briefly, L02 cells and 3T3-L1 cells (1 × 10^4^ per well, 100 *μ*L) were seeded in 96-well plates at 37°C for 24 h in the incubator. Then, the medium was replaced with a fresh 50 *μ*L DMEM medium containing 0.1% DMSO and various concentrations of phenolics (5, 10, 20, 50, 100, 150, and 200 *μ*mol/L). Each concentration sample was repeated three times. The control group only contained 0.1% DMSO. After incubation for 48 h, the medium was discarded. The cells were washed with PBS and then incubated with 100 *μ*L of MTT solution (0.5 mg/mL) for 4 h in the dark. After removing the supernatant, 150 *μ*L of DMSO was added to completely solubilize formazan. The absorbances of wells were measured by a microplate reader with a test wavelength of 570 nm. Cell viability was expressed as a percentage calculated using the following equation: cell viability = [(mean absorbance of each treatment group)/(mean absorbance of control group)] × 100%.

### 2.6. Determination of Intracellular Lipid Content in L02 Cells

To establish intracellular lipid accumulation model, L02 cells were seeded in cell slides of 12-well plates for 12 h to allow cell attachment. A total of 21 wells for each phenolic were assigned into seven groups in triplicate as follows: the control group only contained 0.375% BSA and 0.1% DMSO. The other wells also contained 0.375% BSA and 0.1% DMSO. The model group contained 300 *μ*mol/L PA. The positive control group contained 300 *μ*mol/L PA and 100 *μ*mol/L FFBA. The drug treatment groups contained 300 *μ*mol/L PA and various concentrations of phenolic compounds with 5 *μ*mol/L, 10 *μ*mol/L, 50 *μ*mol/L, and 100 *μ*mol/L, respectively.

The total contents of intracellular lipids were detected by Oil Red O staining as described by Ramirez-Zacarias [29], with minor modifications. After continuous culture for 24 h, L02 cells were fixed in 4% paraformaldehyde for 15 min. The fixed L02 cells were washed with water twice and stained with 0.5% Oil Red O solution in 60 : 40 (v/v) isopropanol/H_2_O for 10 min at 4°C in the dark. Then, the L02 cells were again washed with water and counterstained with hematoxylin for the 30 s. The excess hematoxylin was washed cleanly. The stained L02 cells were stored in glycerol, and oil droplet distribution was captured under an optical microscope. Lipid accumulation relative ratio (LARR) was calculated by using oil area and total area.

### 2.7. Detection of Extracellular Adipokines in 3T3-L1 Cells

To establish an extracellular adipokine secretion abnormality model, adipocyte differentiation of 3T3-L1 cells was implemented with 10% FBS and a mixture of 0.5 mmol/L IBMX, 1.0 *μ*mol/L dexamethasone, and 10 *μ*g/mL insulin. After initiating differentiation for 48 h, the medium was replaced with 10 *µ*g/mL insulin and the cells were continuous cultured in this medium for 96 h. Then, the medium was replaced with DMEM containing 10% FBS each day until the indicated time point. Treated 3T3-L1 cells were cultured in 24-well plates. A total of 12 wells for each phenolic were assigned into four groups with triple parallels as follows: the control group only contained 0.375% BSA and 0.1% DMSO. The other wells also contained 0.375% BSA and 0.1% DMSO. The model group contained 500 *μ*mol/L PA. The drug treatment groups contained 500 *μ*mol/L PA and two different concentrations of the phenolic compound with 10 *μ*mol/L and 20 *μ*mol/L.

After continuous culture for 24 h, the supernatants of 3T3-L1 cells were collected and centrifuged at 10,000 × *g* for 10 min at 4°C to remove solid particles. Then, the expression levels of seven adipokines (adiponectin, IL-6, leptin, MCP-1, resistin, PAI-1, and TNF-*α*) were simultaneously detected using multiplex cytokine kit, according to the manufacturers' protocol. The assays were run in triplicate for each sample, and the data were collected by Luminex-100 system Version 1.7.

### 2.8. Western Blot Analysis

To investigate the molecular mechanisms of CACE in alleviating lipid metabolism disorder, L02 cells were seeded in 6-well plates and incubated for 12 h. A total of 15 wells were assigned into five groups with triple parallels as follows: the control group only contained 0.375% BSA and 0.1% DMSO. The other wells also contained 0.375% BSA and 0.1% DMSO. The model group contained 300 *μ*mol/L PA. The positive control group contained 300 *μ*mol/L PA and 100 *μ*mol/L TUDCA. The drug treatment groups contained two different concentrations of CACE with 10 *μ*mol/L and 100 *μ*mol/L.

The expression levels of key regulated proteins in endoplasmic reticulum (ER) stress and PPARs were determined using western blot analysis. Briefly, after abovementioned treatments for 24 h, cells were harvested and the proteins of cells were extracted with RIPA lysis buffer (1% PMSF) and then centrifuged at 10,000 × *g* for 10 min at 4°C. Protein concentrations in supernatants were determined by a BCA protein assay kit (Beyotime). A twenty-*μ*g of total protein was separated by 12% SDS-PAGE gels and transferred onto polyvinylidene fluoride (PVDF) membranes. PVDF membranes were blocked with 5% free fatty acid milk in 0.5% Tween-20-TBS (TBST) for 40 min at room temperature and incubated in blocking solution containing primary antibodies overnight at 4°C. After washing with TBST, the PVDF membranes were incubated with secondary antibodies conjugated with horseradish peroxidase for 2 h at room temperature. The bound immunoproteins were visualized by ECL reaction, and then the intensities were quantified by Image-Pro Plus software.

### 2.9. Statistical Analysis

The data were expressed as the mean ± standard error mean. The SPSS 19 software was used for statistical analysis. The results were evaluated using Duncan's multiple range test and one-way analysis of variance (ANOVA) and *t*-test. *P* values < 0.05 were considered to indicate statistical significance.

## 3. Results

### 3.1. Phenolic Composition of Propolis Samples

**Figure 1:**
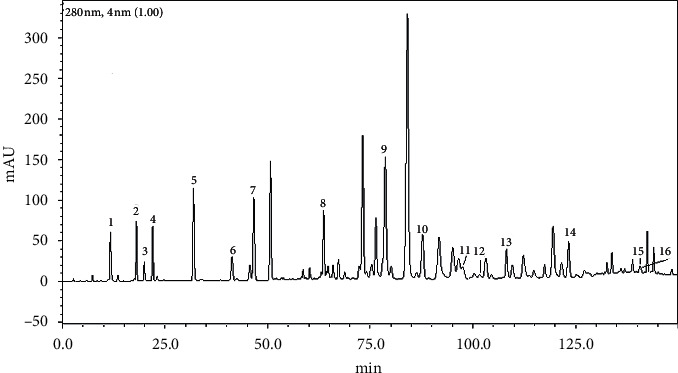
HPLC profiles of poplar-type propolis samples. *Note*. Peak no. (1) caffeic acid (CFA), (2) *p*-coumaric acid (CMA), (3) ferulic acid (FRA), (4) isoferulic acid (IFRA), (5) 3,4-dimethoxycinnamic acid (DMCA), (6) cinnamic acid (CNA), (7) 4-methoxycinnamic acid (MCNA), (8) cinnamylideneacetic acid (CDA), (9) caffeic acid benzyl ester (CABE), (10) caffeic acid phenethyl ester (CAPE), (11) *p*-coumaric acid benzyl ester (CMBE), (12) ferulic acid benzyl ester (FABE), (13) caffeic acid cinnamyl ester (CACE), (14) *p*-coumaric acid cinnamyl ester (CMCE), (15) cinnamic acid cinnamyl ester (CCE), and (16) 4-methoxycinnamic acid cinnamyl ester (MCC).

The phenolic composition of ethanolic extracts from 15 propolis samples was analyzed by HPLC-PDA (Figure 1). Sixteen major phenolics were identified and included 8 phenolic acids and 8 phenolic acid esters, and the contents of these phenolics quantified by external standard are shown in Table 1. As can be seen, the compositions of phenolic acids and esters in all propolis samples looked similar and their contents were obviously different. As a whole, the contents of the phenolic acids were significantly lower than those of phenolic acid esters, constituting approximately 20% of identified phenolics. For phenolic acid esters, the most abundant was CABE, followed by CACE and CMCE. Moreover, it was worthwhile to note that three caffeic acid esters (CAPE, CACE, and CABE) presented relatively high average contents in all propolis samples, accounting for over half of identified phenolics.

### 3.2. Cell Viability of Phenolics

The cell viability of phenolics on L02 cells and 3T3-L1 cells is seen in Figure S1. Almost all of phenolic acids did not have obvious growth-inhibitory activities on both cells, except for 200 *μ*mol/L CMA and FRA-treated L02 cells and 150 *μ*mol/L and 200 *μ*mol/L CDA-treated 3T3-L1 cells. Compared with phenolic acids, phenolic esters presented significant cell toxicities (Figure S2). For example, when treating cells with high concentrations (200 *μ*mol/L) of CAPE, CACE, CABE, and CMCE, only less than half of L02 cells and 3T3-L1 cells were alive. In addition, the tolerance concentration of L02 cells was higher than that of 3T3-L1 cells. According to Figure S2, the L02 cell viability fell to less than 80% after the treatment of 150 *μ*mol/L CAPE, CACE, CABE, and CMCE, compared to 3T3-L1 cells with the treatment of 50 *μ*mol/L CMBE, CAPE, and CACE. Based on these results, the treatment concentrations of phenolics for L02 cells and 3T3-L1 cells were, respectively, 100 *μ*mol/L and 20 *μ*mol/L in the subsequent experiments.

### 3.3. Phenolics Inhibited Intracellular Lipid Accumulation

**Figure 2:**
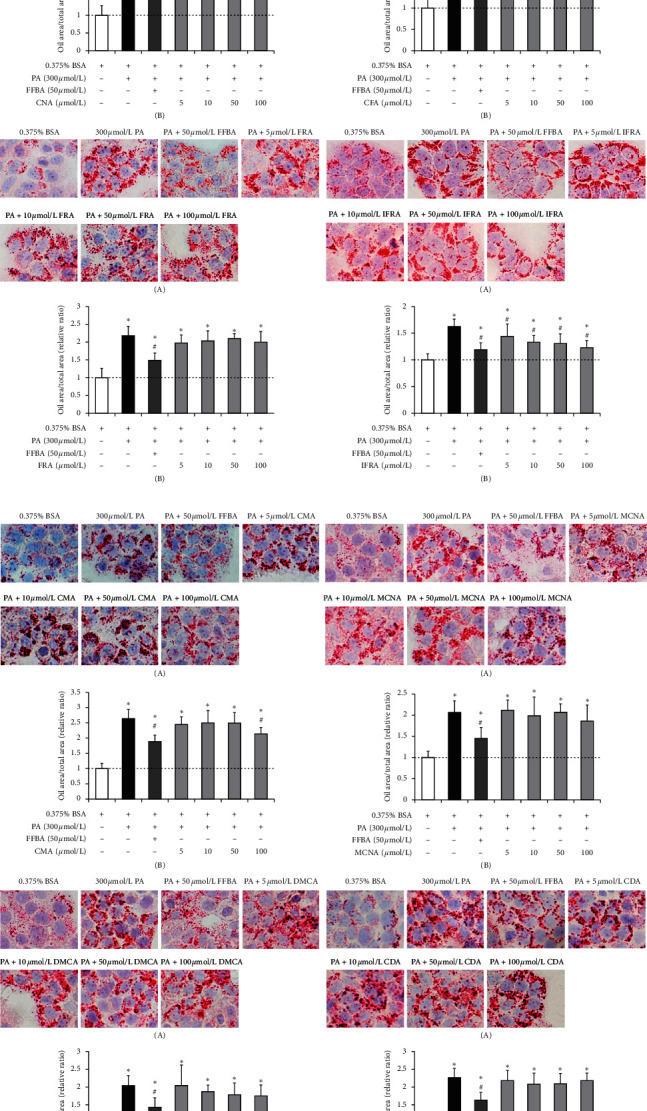
Phenolic acids reduce intracellular lipid contents in L02 cells. *Note*. (1) The cells were treated with palmitic acid (PA) in the absence or presence of different doses of phenolic acids for 24 h. (A) Images of cells were captured by microscope at 400 × original magnification showing lipid accumulation in cells sustained by Oil Red O. (B) Lipid accumulation relative ratio. (2) 0.375% bull serum albumin (BSA) treatment is the control group; 300 *μ*mol/L PA treatment is the model group; 300 *μ*mol/L PA and 50 *μ*mol/L fenofibric acid (FFBA) treatment is the positive control group. 300 *μ*mol/L PA and various concentrations of phenolic compound treatment are drug treatment groups. Significant differences (*P* < 0.05) from the control group are marked with ∗ and from the model group are marked with #. (3) CNA: cinnamic acid; CFA: caffeic acid; FRA: ferulic acid; IFRA: isoferulic acid; CMA: *p*-coumaric acid; MCNA: 4-methoxy cinnamic acid; DMCA: 3, 4-dimethoxy cinnamic acid; CDA: cinnamylideneacetic acid.

**Figure 3:**
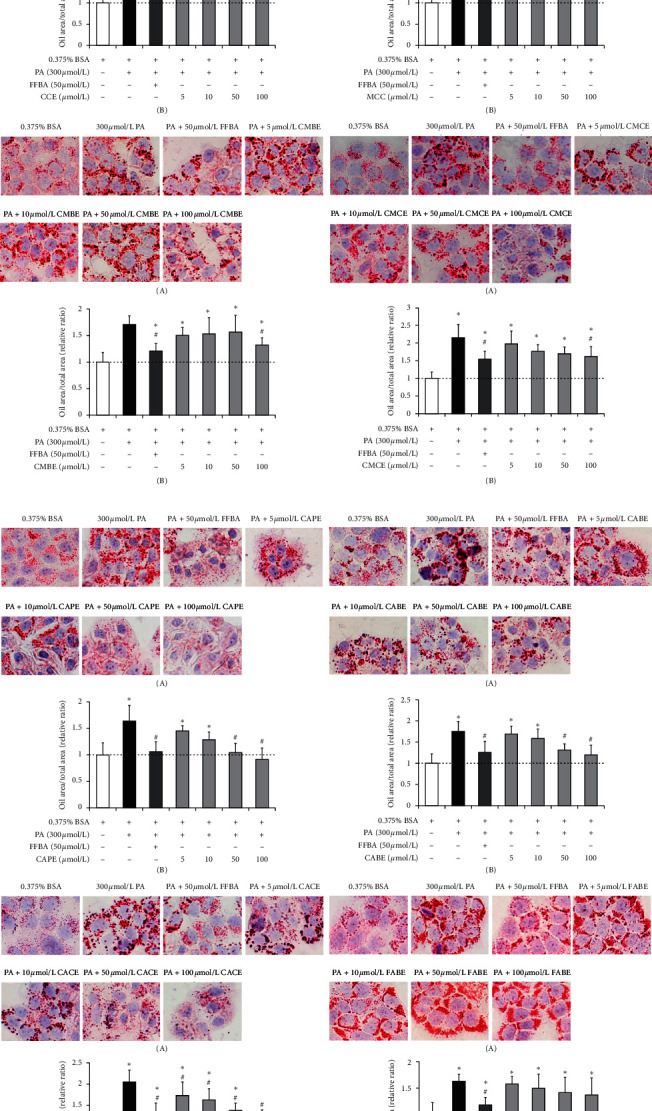
Phenolic esters reduce intracellular lipid contents in L02 cells. *Note*. (1) The cells were treated with palmitic acid (PA) in the absence or presence of different doses of phenolic esters for 24 h. (A) Images of cells were captured by microscope at 400 × original magnification showing lipid accumulation in cells stained by Oil Red O. (B) Lipid accumulation relative ratio. (2) 0.375% bull serum albumin (BSA) treatment is the control group; 300 *μ*mol/L PA treatment is the model group; 300 *μ*mol/L PA and 50 *μ*mol/L fenofibric acid (FFBA) treatment is the positive control group. 300 *μ*mol/L PA and various concentrations of phenolic compound treatment are drug treatment groups. Significant differences (*P* < 0.05) from the control group are marked with ∗ and from the model group are marked with #. (3) CCE: cinnamic acid cinnamyl ester; MCC: 4-methoxy cinnamic acid cinnamyl ester; CMBE: *p*-coumaric acid benzyl ester; CMCE: *p*-coumaric acid cinnamyl ester; CAPE: caffeic acid phenethyl ester; CABE: caffeic acid benzyl ester; CACE: caffeic acid cinnamyl ester; FABE: ferulic acid benzyl ester.

The abilities of phenolics to prevent lipid accumulation were investigated by determining intracellular lipid contents using Oil Red O staining. Figures 2 and 3 show the images and LLARs of intracellular lipid accumulation after the 24 h treatment of L02 cells with PA (the model group) and cotreatment of L02 cells with PA and different phenolics. FFBA, a common lipid-lowering drug, is often used as a positive control in many similar trials [30]. As can be seen, the cells treated with 50 *μ*mol/L FFBA decreased an average of 30% in lipid accumulation, compared with the model group. For phenolic acids, only CMA and IFRA reduced lipid accumulation levels of 20% and 22% at the treatment concentrations of 100 *μ*mol/L (Figure 2). In contrast, phenolic esters presented apparently inhibitory effects, particularly three caffeic acid esters. According to Figure 3, when the treatment concentrations reached 100 *μ*mol/L, these three caffeic acid esters (CAPE, CACE, and CABE) significantly diminished 48%, 44%, and 42% of lipid accumulation levels, respectively. The lipid-lowering activities of phenolics may be triggered by their powerful abilities against free radicals [31]. These “radical scavengers” provide hydrogen to free radicals of lipid compounds and lead themselves to transform into phenolic hydroxyl radicals. The phenolic hydroxyl radicals can lower the transfer speed of auto-oxidation chain reaction and exert an important function in inhibiting lipid peroxidation [32]. Furthermore, our results also indicated that phenolic esters exhibit better decreasing levels in intracellular lipid accumulation than phenolic acids, especially three caffeic acid esters. We suspect that the prominent effects of three caffeic acid esters may be due to their similar structures, containing two phenolic hydroxyl groups on the aromatic ring as donor substituents to increase the activity of hydrogen atoms and to lower steric hindrance of phenol hydroxyl radical [33]. Based on the above results, CAPE, CACE, and CABE were selected in the subsequent adipokine experiment.

### 3.4. Three Caffeic Acid Esters Amended the Secretion Abnormalities of Extracellular Adipokines

**Figure 4:**
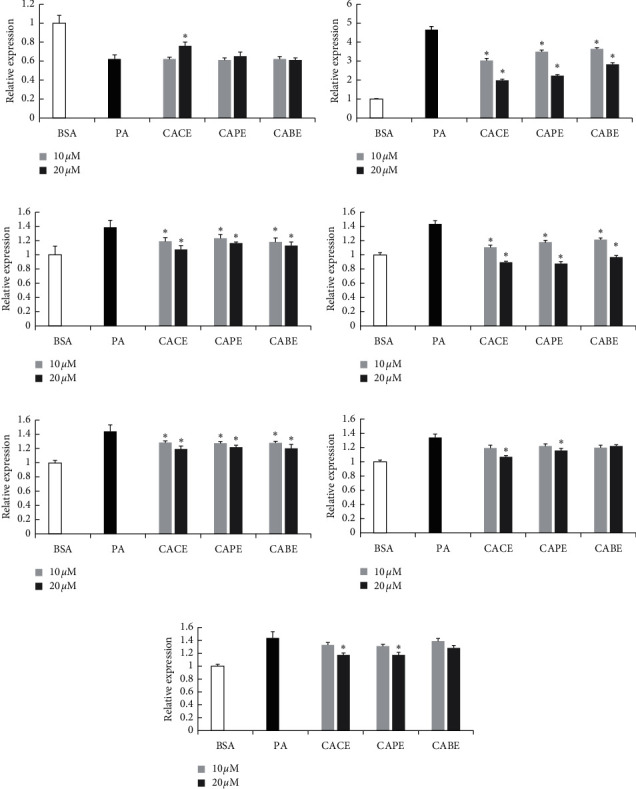
Effects of caffeic acid cinnamyl ester (CACE), caffeic acid phenethyl ester (CAPE), and caffeic acid benzyl ester (CABE) on the expressions of seven adipocytes in differentiated 3T3-L1 cells induced by palmitic acid (PA). (a) Adiponectin. (b) IL-6. (c) Leptin. (d) MCP-1. (e) Resistin. (f) PAI-1. (g) TNF-*α*. *Note*. 0.375% bull serum albumin (BSA) treatment is the control group; 500 *μ*mol/L PA treatment is the model group. 500 *μ*mol/L PA and two different concentrations of the phenolic compound treatment are drug treatment groups. All values represent the mean of triplicate determinations ± SD. Significant differences (*P* < 0.05) from the model group are marked with ^∗^.

Lipid metabolism disorder involves not only in intracellular lipid accumulation but in extracellular adipokine secretion abnormalities [34]. Adipokines, primarily secreted by adipose tissue cells, are bioactive molecules. Numerous studies have shown that these molecules can regulate several physiological functions such as energy balance, insulin sensitization, appetite regulation, inflammatory response, and vascular homeostasis [35, 36]. Thus, to investigate whether CAPE, CACE, and CABE can affect adipokine secretion, the expression levels of seven adipokines were measured in differentiated 3T3-L1 cells by the treatment of PA (model group) and the respective cotreatment of PA and CAPE, CACE, and CABE, using multiplex cytokine kit. As revealed in Figure 4, compared with the model group, CACE with 20 *μ*mol/L treatment significantly amended the levels of all seven adipokines through downregulating the expression of leptin, resistin, IL-6, MCP-1, PAI-1, and TNF-*α*, and upregulating the expression of adiponectin. In contrast, CAPE treatment did not affect the expression of adiponectin. Similarly, CABE treatment had no influence on adiponectin and PAI-1. Moreover, CACE seemed to increase regulation effects dependent on dose. As a whole, CACE presented a more prominent effect on ameliorating the secretion abnormalities of extracellular adipokines than the other two caffeic acid esters. Therefore, CACE was used to further assess the possible molecular mechanisms on alleviating lipid metabolism disorder.

### 3.5. CACE Regulated ER Stress and PPARs

**Figure 5:**
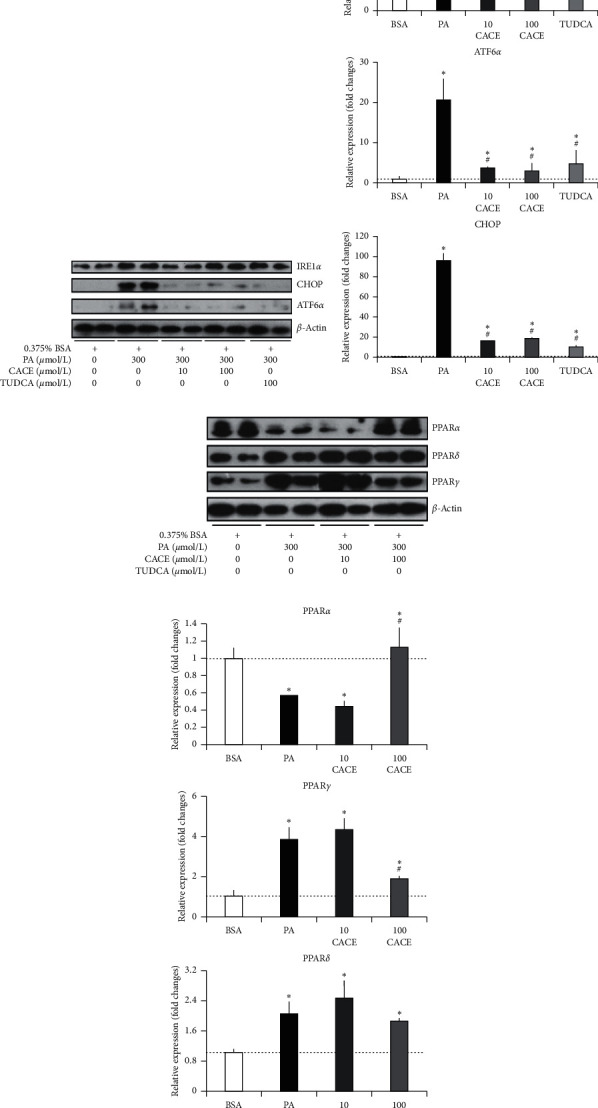
Effect of caffeic acid cinnamyl ester (CACE) on the expressions of ER stress proteins and PPARs in palmitic acid (PA)-stimulated L02 cells. *Note*. (1) (a), (c) western blot analysis of IREI*α*, CHOP, and ATF6*α* protein-associated ER stress pathways, and PPAR*α*, PPAR*δ*, and PPAR*γ* proteins; (b), (d) the relative expressions of IREI*α*, CHOP, ATF6*α*, PPAR*α*, PPAR*δ*, and PPAR*γ* proteins. 2. 0.375% bull serum albumin (BSA) treatment is the control group; 300 *μ*mol/L PA treatment is the model group; 300 *μ*mol/L PA and 100 *μ*mol/L tauroursodesoxycholic acid (TUDCA) treatment is the positive control group. Two different concentrations of CACE treatment are drug treatment groups. All values represent the mean of triplicate determinations ± SD. Significant differences (*P* < 0.05) from the control group are marked with ∗ and significant differences (*P* < 0.05) from the model group are marked with #.

The molecular mechanisms of CACE in alleviating lipid metabolism disorder were investigated by detecting associated protein expression of ER stress and PPARs using western blot analysis. TUDCA is a bile acid derivative as a chemical chaperone to ameliorate ER stress and was used as a positive control in this research [37]. As can be seen in Figures 5(a) and 5(b), after L02 cells were induced by PA and then treated with 10 or 100 *μ*mol/L CACE, the expression levels of CHOP and ATF6*α* remarkably decreased (*P* < 0.05), whereas IRE1*α* did not obviously change. These results were similar to those treated by 100 *μ*mol/L TUDCA as a positive control. Moreover, according to Figures 5(c) and 5(d), CACE treatment with 100 *μ*mol/L significantly upregulated and recovered the PPAR*α* expression (*P* < 0.05) and downregulated PPAR*γ* expression (*P* < 0.05), while only PA treatment (model group) significantly downregulated PPAR*α* expression and upregulated PPAR*δ* and PPAR*γ* expressions.

## 4. Discussion

Lipid metabolism disorder refers to dyslipidemia in the plasma and is one of the high-risk factors for many diseases, including obesity, nonalcoholic fatty liver, and cardiovascular diseases. Lipid metabolism disorder has become a serious threat to human health [38]. To prevent and treat lipid metabolism disorder, great efforts are being made to search effective therapeutic interventions. Recently, propolis was discovered with significant lipid-regulating activity and remedying lipid metabolic diseases [39, 40]. However, these studies only focused on extracts or flavonoids from propolis, and less research is conducted to evaluate the effects of phenolic acids and esters from propolis on the regulation of lipid metabolism. In this study, we investigated the lipid-lowering activities of phenolics identified in poplar-type propolis and the molecular mechanism of alleviating lipid metabolism disorder.

We find that all propolis samples displayed similar chemical compositions and abundant contents of phenolic acids and esters. The total contents of phenolic esters are significantly higher than those of phenolic acids (Table 1). These results are consistent with the previous research that phenolic esters and flavonoids were the main compound classes found in poplar-type propolis [41]. In addition, other previous studies have noted that phenolic acid and ester compounds play a positive role in amending lipid metabolism. For example, several studies have shown that CAPE has antioxidant, anti-inflammatory, and cytostatic properties and hepatorenal protective effects against the cytotoxic injuries linked to metabolic syndrome and vascular diseases [42–45]. CAPE can also reduce the activation of the nuclear factor *κ*B pathway in high-fat diet-induced obesity mice [46]. Ferulic acid can affect the glucose and lipid homeostasis in HFD-induced obese mice via modulating the expression of lipogenic and gluconeogenic genes in liver tissues [47]. Therefore, the lipid-regulating activities of more phenolic acids and esters are worth further research.

Our results reveal that all treatments of 8 phenolic acids and 8 phenolic esters reduced intracellular lipid accumulation in PA-stimulated L02 cells in different degrees (Figures 2 and 3) without producing cytotoxic effects (Figures S1 and S2). The lipid-lowering activities of phenolic esters were apparently higher than those of phenolic acids. Specifically, we find that three caffeic acid esters (CAPE, CACE, and CABE) with 100 *μ*mol/L markedly reduced the amounts of lipid accumulation almost to the basal level (Figure 3). On the other hand, adipokines, as a kind of inflammatory cytokines released by adipocytes, can exert their unique biological activities and influence several physiological processes concerning immunity and metabolism. The adipokine dysregulation may also be linked to lipid metabolism disorder in adipose tissue [48]. Prior research has shown that most of adipokine secretion was increased in obese adipose tissue as proinflammatory adipokines; for example, TNF-*α*, IL-6, PAI-1, leptin, resistin, and MCP-1 can promote lipid metabolic diseases [49]. In contrast, adipose tissue from lean state preferentially secretes such anti-inflammatory adipokines as adiponectin [49]. The present work evaluated the regulation effects of CAPE, CACE, and CABE on the secretion of abovementioned adipokines in differentiated 3T3-L1 cells induced by PA. We find that 20 *μ*mol/L CACE can significantly regulate the expression levels of all seven adipokines with downregulating TNF-*α*, IL-6, PAI-1, MCP-1, leptin, and resistin expression and upregulating adiponectin expression (Figure 4). Overall, our study reveals a novel finding that CACE presents prominent lipid-regulating activities on both preventing intracellular lipid accumulation and amending extracellular adipokine secretion abnormalities.

ER stress refers to a condition of accumulating unfolded or misfolded proteins in the ER when cells suffer various pathophysiologic states [50]. Much evidence has suggested that ER stress is a mediator of impaired lipid metabolism, thereby leading to various lipid metabolism diseases, for example, fatty liver and atherosclerosis [51]. To alleviate ER stress, cells initiate the activation of various protective strategies, collectively termed the unfolded protein response (UPR). The UPR mechanism consists of three distinct signaling pathways: (a) protein kinase RNA-like endoplasmic reticulum kinase (PERK); (b) activating transcription factor 6 (ATF6); and (c) inositol-requiring protein 1 (IRE1) [52]. The three pathways contain three key marker proteins, named as CHOP, ATF6*α*, and IREI*α*, respectively [53]. In our study, the expression levels of CHOP, ATF6*α*, and IREI*α* were obviously upregulated when PA induced L02 cells, indicating remarkable URP production. After CACE or TUDCA treatment (100 *μ*mol/L), the levels of CHOP and ATF6*α* were significantly reduced (*P* < 0.05), while IREI*α* expression was nearly unchanged (Figures 5(a) and 5(b)). CHOP is an apoptosis signaling molecule induced by ER stress and plays a vital role in cell apoptosis [54]. In addition, ATF6*α* deficiency can improve insulin sensitivity and restrain the development of insulin resistance. The improved insulin sensitivity in ATF6*α*-deficient DO mice can be due to partial suppression in the development of hypertriglyceridemia [55]. Therefore, we suspect that CACE can suppress the activation of PERK- and ATF6-associated ER stress pathways, leading to protect cells against ER stress-induced cell apoptosis and improve cells to recover insulin resistance.

PPARs are ligand-activated transcription factors and belong to the superfamily of nuclear hormone receptors to regulate a plethora of expression of genes involved in metabolism processes [56]. Similar to ER stress, PPARs are also a metabolic switches to respond to changes in cellular lipid status and play crucial roles to achieve the balance of lipid metabolism. The PPAR family consists of three ligand-activated nuclear receptors, including PPAR*α*, PPAR*β*/*δ*, and PPAR*γ*. Each of them displays a unique pattern of tissue-specific expression to reflect their distinctive functions [57]. There is evidence that PPAR*α* can function as a lipid sensor to recognize and respond to the influx of fatty acids by stimulating the transcription of numerous genes related to lipid metabolism, including mitochondrial *β*-oxidation, peroxisomal *β*-oxidation, fatty acid uptake and binding, and lipoprotein assembly and transport [58]. Unlike PPAR*α*, PPAR*γ* is an important mediator for adipogenesis of adipose tissue to control the expression of some genes related to adipocyte differentiation [59]. Our results indicate that, compared with the model group, CACE treatment (100 *μ*mol/L) significantly upregulated the expression of PPAR*α* (*P* < 0.05), leading to increased transcription activation of genes linked to fatty acid catabolism (Figures 5(c) and 5(d)). In addition, CACE treatment (100 *μ*mol/L) can significantly downregulate the level of PPAR*γ* (*P* < 0.05) to inhibit lipid anabolism. In other words, CACE treatment could promote lipid metabolism trend toward catabolism. The present results seem to concur with the previous report that the diet supplemented with 0.5% propolis can decrease fat accumulation in high-fat-fed rats through regulating the expression levels of PPAR*α* and PPAR*γ* in adipose tissue [40]. Overall, this seems to be the first report that CACE can alleviate lipid metabolism disorder in PA-induced L02 cells through suppressing ER stress and activating PPARs.

## 5. Conclusion

Our results reveal that phenolic esters in poplar-type propolis are more abundant and have higher lipid-lowering activities than phenolic acids, particularly for CAPE, CACE, and CABE. We also report here for the first time that CACE has outstanding properties to reduce intracellular lipid accumulation and regulate extracellular adipokines in PA-stimulated cells. Moreover, CACE can alleviate lipid metabolism disorder through inhibiting ER stress via PERK and ATF6 signaling pathways and activating PPARs with upregulating PPAR*α* expression and downregulating PPAR*γ* expression. The current study seems to provide a reliable evidence for developing propolis nutraceuticals directed at lipid metabolism disorder.

## Figures and Tables

**Table 1:** The contents of phenolics identified in poplar-type propolis (mg/g).

Phenolics	Three samples from Anhui Province	Three samples from Hubei Province	Three samples from Hunan Province	Three samples from Shandong Province	Three samples from Zhejiang Province	Average
Caffeic acid	1.85 ± 0.11	2.58 ± 0.13	6.52 ± 0.88	1.28 ± 0.11	3.49 ± 0.39	2.93
*p*-Coumaric acid	1.81 ± 0.14	1.25 ± 0.10	2.07 ± 0.27	5.45 ± 0.67	1.46 ± 0.18	2.31
Ferulic acid	0.96 ± 0.08	1.15 ± 0.09	2.36 ± 0.25	0.98 ± 0.11	1.44 ± 0.09	1.31
Isoferulic acid	2.63 ± 0.18	1.26 ± 0.12	1.85 ± 0.10	0.59 ± 0.04	1.88 ± 0.27	1.81
3,4-Dimethoxycinnamic acid	2.51 ± 0.15	1.77 ± 0.15	3.99 ± 0.34	0.70 ± 0.08	2.81 ± 0.31	2.38
Cinnamic acid	0.54 ± 0.05	0.16 ± 0.02	0.18 ± 0.04	0.54 ± 0.04	0.17 ± 0.04	0.36
4-Methoxycinnamic acid	0.79 ± 0.10	0.24 ± 0.05	0.24 ± 0.05	—	0.40 ± 0.08	0.41
Cinnamylidene acetic acid	3.13 ± 0.28	1.95 ± 0.14	0.86 ± 0.07	1.49 ± 0.14	1.14 ± 0.14	1.95
Caffeic acid benzyl ester	17.76 ± 2.11	14.60 ± 1.56	13.31 ± 1.23	4.82 ± 0.55	8.86 ± 1.20	12.85
Caffeic acid phenethyl ester	9.39 ± 1.25	7.74 ± 0.98	10.83 ± 1.87	1.73 ± 0.22	9.52 ± 1.13	8.10
*p*-Coumaric acid benzyl ester	1.35 ± 0.18	3.65 ± 0.35	7.60 ± 0.11	8.57 ± 1.04	0.77 ± 0.11	3.88
Ferulic acid benzyl ester	4.93 ± 0.53	4.94 ± 0.47	7.59 ± 0.14	—	5.39 ± 0.45	4.63
Caffeic acid cinnamyl ester	9.09 ± 1.32	7.60 ± 0.88	15.57 ± 1.04	10.28 ± 1.56	10.52 ± 1.16	10.36
*p*-Coumaric acid cinnamyl ester	6.68 ± 0.97	4.76 ± 0.41	7.96 ± 0.93	25.00 ± 3.89	5.54 ± 0.41	9.44
Cinnamic acid cinnamyl ester	0.62 ± 0.04	0.42 ± 0.07	0.62 ± 0.08	5.28 ± 0.58	0.38 ± 0.06	1.32
4-Methoxycinnamic acid cinnamyl ester	2.65 ± 0.15	2.86 ± 0.31	3.02 ± 0.33	—	2.67 ± 0.32	2.31

Note: —, not detected.

## Data Availability

The data used to support the findings of this study are available from the corresponding author upon request.

## Abbreviations

CFA: Caffeic acid
CMA: *p*-Coumaric acid
FRA: Ferulic acid
IFRA: Isoferulic acid
DMCA: 3,4-Dimethoxycinnamic acid
CNA: Cinnamic acid
MCNA: 4-Methoxycinnamic acid
CDA: Cinnamylideneacetic acid
CABE: Caffeic acid benzyl ester
CAPE: Caffeic acid phenethyl ester
CCE: Cinnamic acid cinnamyl ester
FABE: Ferulic acid benzyl ester
CMBE: *p*-Coumaric acid benzyl ester
CACE: Caffeic acid cinnamyl ester
MCC: 4-Methoxycinnamic acid cinnamyl ester
CMCE: *p*-Coumaric acid cinnamyl ester
DMSO: Dimethyl sulfoxide
BCA: Bicinchoninic acid
MTT: 3-(4,5-Dimethyl-2-thiazolyl)-2,5-diphenyl-2-H-tetrazolium bromide
PA: Palmitic acid
FFBA: Fenofibric acid
TUDCA: Tauroursodesoxycholic acid
IBMX: Isobutyl methyl xanthine
FBS: Fetal bovine serum
BSA: Bull serum albumin
DMEM: Dulbecco's modified eagle medium
PBS: Phosphate-buffered saline
IL-6: Interleukin-6
MCP-1: Monocyte chemoattractant protein-1
PAI-1: Plasminogen activator inhibitor-1
TNF-*α*: Tumor necrosis factor-alpha
CHOP: Proapoptotic CCAAT-enhancer-binding protein homologous protein
PPARs: Peroxisome proliferator-activated receptors
PMSF: Phenylmethylsulfonyl fluoride
ER: Endoplasmic reticulum
PVDF: Polyvinylidene fluoride
UPR: The unfolded protein response
PERK: The protein kinase RNA-like ER kinase
ATF6: Activating transcription factor 6
IRE1: Inositol-requiring protein 1.

## Supplementary Materials

Supplementary Materials Figure S1: the cell viability of phenolic acids at different concentrations on L02 cells (a) and 3T3-L1 cells (b). *Note*. (1) The results shown are representative of three independent experiments. All values represent the mean of triplicate determinations ± SD. Significant differences (*P* < 0.05) from control are marked with ∗. (2) CNA: cinnamic acid; CFA: caffeic acid; FRA: ferulic acid; IFRA: isoferulic acid; CMA: *p*-coumaric acid; MCNA: 4-methoxy cinnamic acid; DMCA: 3,4-dimethoxy cinnamic acid; CDA: cinnamylideneacetic acid. Figure S2: the cell viability of phenolic esters at different concentrations on L02 cells (a) and 3T3-L1 cells (b). *Note*. (1) The results shown are representative of three independent experiments. All values represent the mean of triplicate determinations ± SD. Significant differences (*P* < 0.05) from control are marked with ∗. (2) CCE: cinnamic acid cinnamyl ester; MCC: 4-methoxy cinnamic acid cinnamyl ester; CMBE: *p*-coumaric acid benzyl ester; CMCE: *p*-coumaric acid cinnamyl ester; CAPE: caffeic acid phenethyl ester; CABE: caffeic acid benzyl ester; CACE: caffeic acid cinnamyl ester; FABE: ferulic acid benzyl ester.
