# Supplementary figures and images for: Phenolic Compounds of Propolis Alleviate Lipid Metabolism Disorder
Source: Evid Based Complement Alternat Med. 2021 Feb 20;2021:7615830. doi: 10.1155/2021/7615830 (PMC7914084; doi:10.1155/2021/7615830)

a

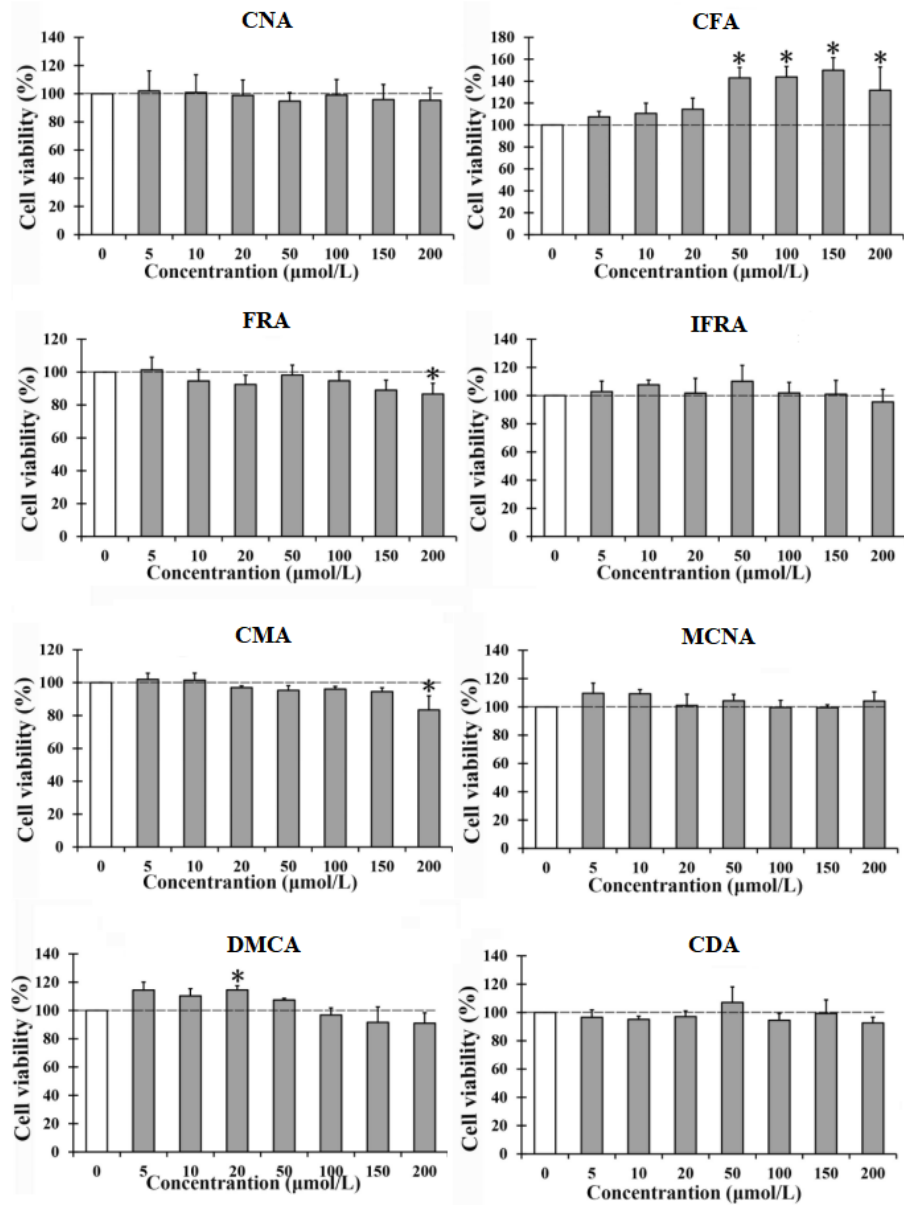

b

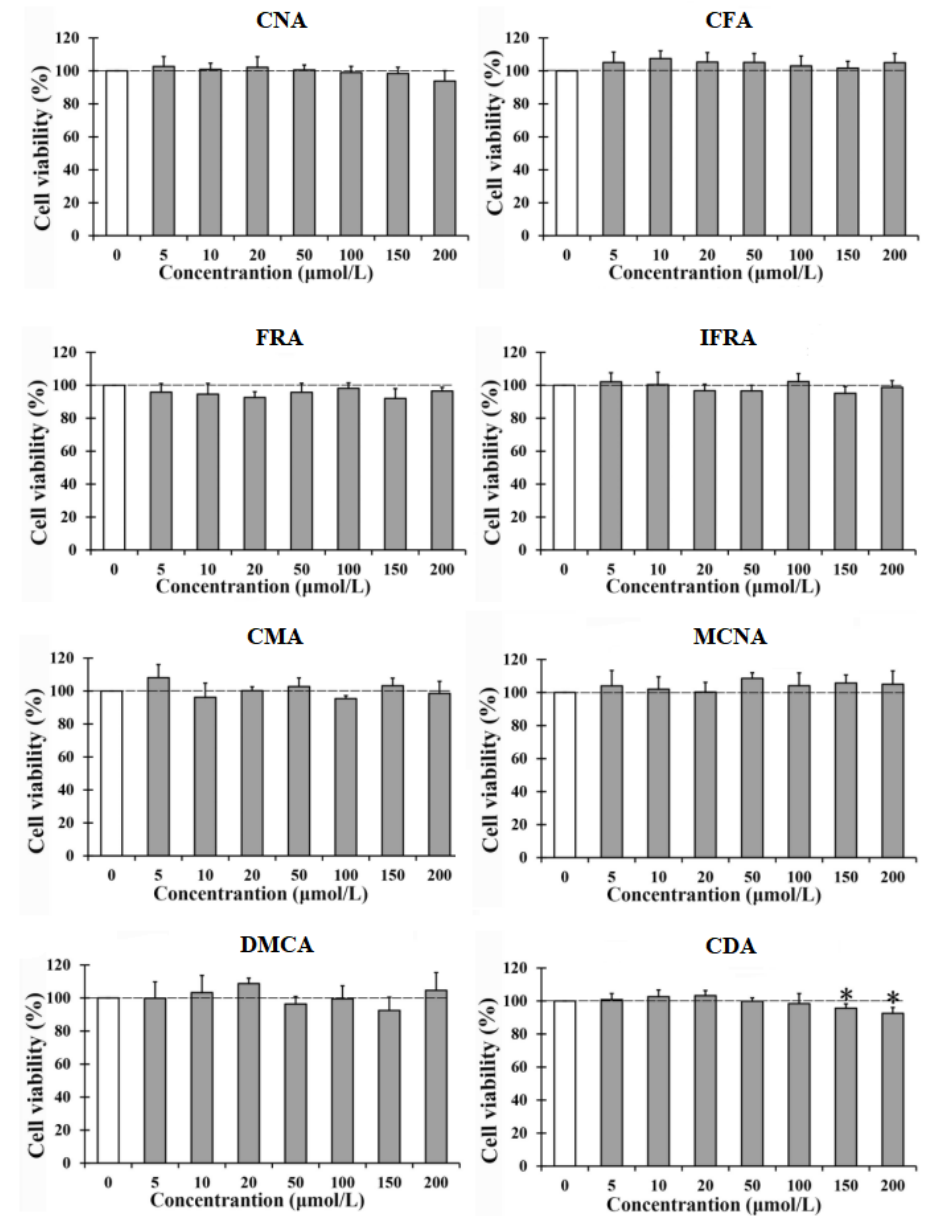

Supplement: Supplementary Materials — Figure S1: the cell viability of phenolic acids at different concentrations on L02 cells (a) and 3T3-L1 cells (b). Note. (1) The results shown are representative of three independent experiments. All values represent the mean of triplicate determinations ± SD. Significant differences (P < 0.05) from control are marked with ∗. (2) CNA: cinnamic acid; CFA: caffeic acid; FRA: ferulic acid; IFRA: isoferulic acid; CMA: p-coumaric acid; MCNA: 4-methoxy cinnamic acid; DMCA: 3,4-dimethoxy cinnamic acid; CDA: cinnamylideneacetic acid. Figure S2: the cell viability of phenolic esters at different concentrations on L02 cells (a) and 3T3-L1 cells (b). Note. (1) The results shown are representative of three independent experiments. All values represent the mean of triplicate determinations ± SD. Significant differences (P < 0.05) from control are marked with ∗. (2) CCE: cinnamic acid cinnamyl ester; MCC: 4-methoxy cinnamic acid cinnamyl ester; CMBE: p-coumaric acid benzyl ester; CMCE: p-coumaric acid cinnamyl ester; CAPE: caffeic acid phenethyl ester; CABE: caffeic acid benzyl ester; CACE: caffeic acid cinnamyl ester; FABE: ferulic acid benzyl ester. [file 7615830.f1.zip › 7615830.f1/Fig.S1 (1).pdf]

**a**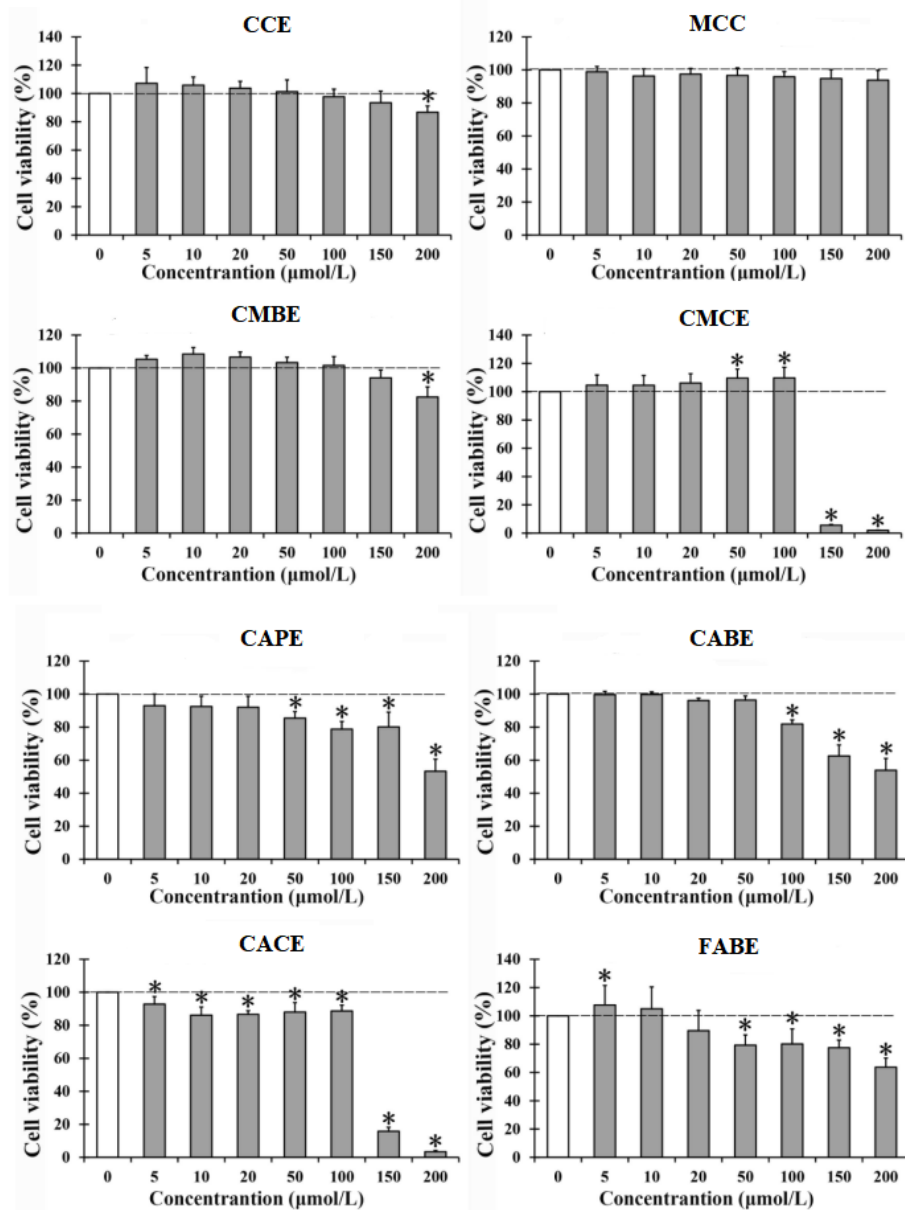**b**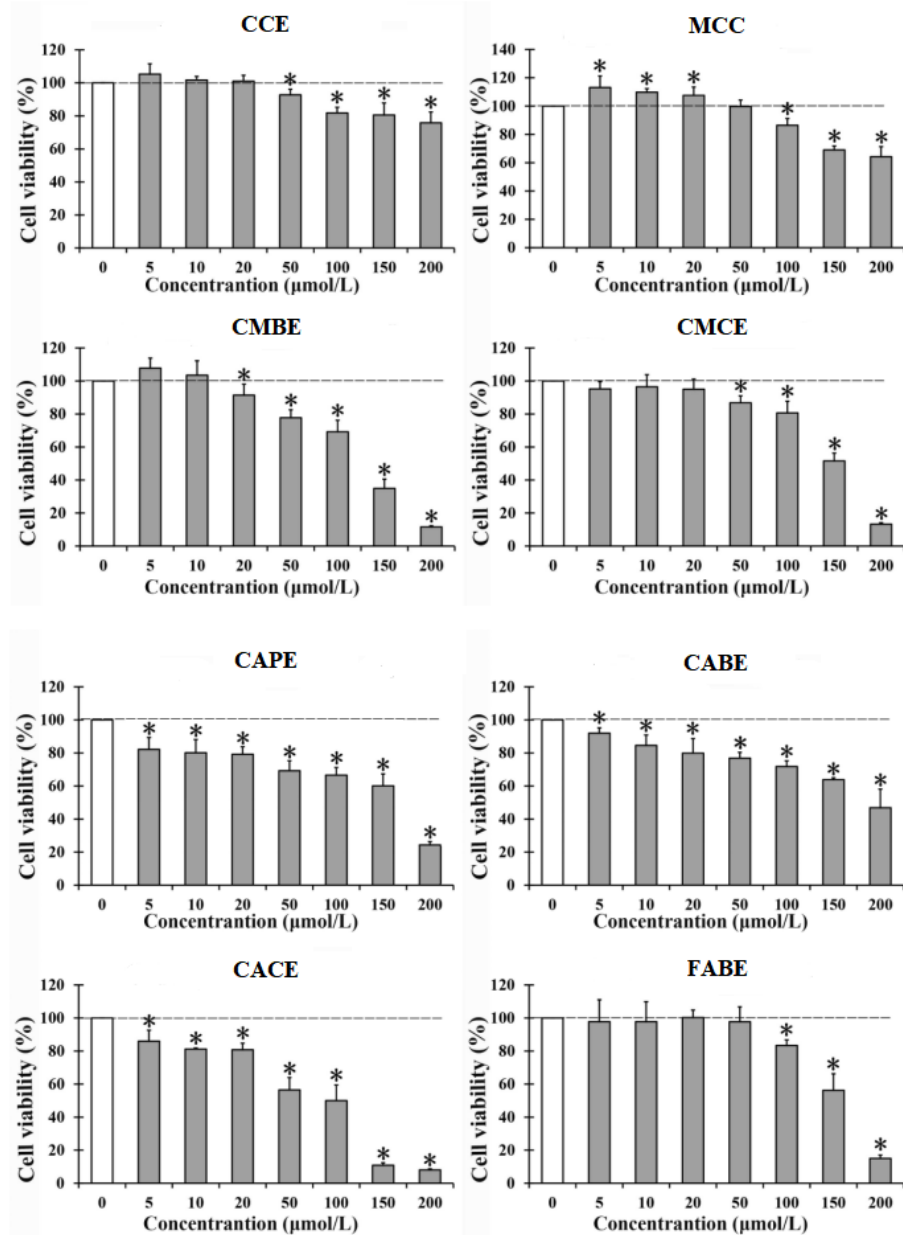

Supplement: Supplementary Materials — Figure S1: the cell viability of phenolic acids at different concentrations on L02 cells (a) and 3T3-L1 cells (b). Note. (1) The results shown are representative of three independent experiments. All values represent the mean of triplicate determinations ± SD. Significant differences (P < 0.05) from control are marked with ∗. (2) CNA: cinnamic acid; CFA: caffeic acid; FRA: ferulic acid; IFRA: isoferulic acid; CMA: p-coumaric acid; MCNA: 4-methoxy cinnamic acid; DMCA: 3,4-dimethoxy cinnamic acid; CDA: cinnamylideneacetic acid. Figure S2: the cell viability of phenolic esters at different concentrations on L02 cells (a) and 3T3-L1 cells (b). Note. (1) The results shown are representative of three independent experiments. All values represent the mean of triplicate determinations ± SD. Significant differences (P < 0.05) from control are marked with ∗. (2) CCE: cinnamic acid cinnamyl ester; MCC: 4-methoxy cinnamic acid cinnamyl ester; CMBE: p-coumaric acid benzyl ester; CMCE: p-coumaric acid cinnamyl ester; CAPE: caffeic acid phenethyl ester; CABE: caffeic acid benzyl ester; CACE: caffeic acid cinnamyl ester; FABE: ferulic acid benzyl ester. [file 7615830.f1.zip › 7615830.f1/Fig.S2 (1).pdf]
